# A glossary for the cross-disciplinary study of numerals

**DOI:** 10.1098/rstb.2025.0321

**Published:** 2025-10-20

**Authors:** Jean-Charles Pelland

**Affiliations:** ^1^ Department of Psychosocial Science, University of Bergen, Bergen, Hordaland 5015, Norway

**Keywords:** numerals, number, glossary

*This terminological framework was developed for this Special Issue with the aim of minimizing potential confusion caused by inconsistent use of central terms in the study of numeration systems across academic disciplines—especially the term ‘base’ and its variants—as well as helping readers with some of the basic terms used in the study of numerals*.

  *Addend* In the operation of addition, the addend is the number that is added *to* another number. So, in *x* + *y* = *z*, *y* is the addend (*x* is the augend and *z* is the sum).

*Anchor* (*or Compositional Anchor*) An anchor is a number conventionally used as a counting unit, via either an arithmetical operation or other compositional operation, as manifested in the use of special representational vehicles. In a non-atomic numeral, the anchor is the number *to which* one or more arithmetical operations are applied to obtain the value of the non-atomic numeral. For example, 10 would be the anchor in ‘fourteen’ since the symbol-formation rule here is to combine a morpheme for 10 (i.e. ‘teen’) with a morpheme for a number added to 10 (i.e. ‘four’) to form the non-atomic numeral whose value is 10 + 4. Special cases of anchors are *bases*, which are anchors whose powers are given special representational status within a numeration system. Anchors can be qualified in terms of both their frequency of use and the arithmetical operation(s) they undergo, as detailed in other entries in this glossary. In principle, any arithmetical operation can be implemented by an anchor, though in practice this is usually addition, multiplication, exponentiation or a combination of these. For example, an anchor can be both multiplicative and additive, and any exponential anchor can be both multiplicative and additive as well. Thus, 10 is a multiplicative–additive anchor in English ‘twenty-nine’, since we obtain the value of the numeral by multiplying 10 by 2 and then adding 9 to that value. The value of composite numerals containing more than two numerals can be obtained via nested anchors, each being the number to which an arithmetical operation is applied. The argument of the arithmetical operation applied to the anchor is called the *compositional input,* or *input*. ‘Anchor’ is a term introduced in this special issue to talk about any number that is used in obtaining the value of non-atomic numerals in a way that applies not just to bases, but also to the value of compositional elements whose use is either restricted in scope, irregular, unsystematic, or somehow seems like a significant departure from how bases are described in the literature on notation and in mathematics. The advantage of introducing this new term is to allow ‘base’ to keep some of its usual associations of productivity and regularity (especially concerning bases in notational systems), while highlighting the fact that bases are not the only way of composing non-atomic numerals. For more on anchors, see the contributions to this special issue by Barlow [1] and by Pelland [2].

*Anchor, additive* An additive anchor is a number *to which* another number is added to obtain the value of a composite numeral. For example, a system glossed as follows would have 5 as an additive anchor in the numerals highlighted in bold: {1, 2, 3, 4, 5, **5+1**, **5+2**, **5+3**, **5+4**, **5+5**, **5+(5+1**), **5+(5+2**) …}. In English, 10 is used as an additive anchor in numerals for 13−19. (Between 20 and 99, English uses 10 as a multiplicative anchor for decade numerals and as a multiplicative–additive anchor for other numerals.)

*Anchor, exponential* An exponential anchor is the smallest number whose powers are used as anchors in a numeration system, as indicated by these powers being denoted by atomic numerals or other special representational vehicles. In other words, exponential anchors are bases. For example, a system glossed as follows would have 5 as an exponential and exponential–additive anchor (highlighted in bold) as well as an additive and multiplicative–additive anchor (not in bold): {1, 2, 3, 4, 5, 5+1, 5+2, 5+3, 5+4, 2⋅5, (2⋅5)+1, (2⋅5)+2 … **FivePower2**, **FivePower2+1** …}. In English, words like ‘hundred’, ‘thousand’ and ‘million’ use 10 as an exponential anchor.

*Anchor, hapax* A hapax anchor is a compositional anchor used in obtaining the value of only one non-atomic numeral within a determined numerical range. A useful range to determine the status of an anchor in non-restricted systems is the *primary counting cycle* (see *counting cycle, primary* in this glossary). Most hapax anchors are also sporadic anchors. In principle, any arithmetical operation can be used by a hapax anchor, though in practice this is usually addition or multiplication. For example, a system glossed as follows uses 5 as a hapax anchor in the numeral for 8 (highlighted in bold): {1, 2, 3, 4, 5, 6, 7, **5+3**, 9, 10, 11 …}. Another example is the Welsh lexical numeral for 18, ‘deunaw’, glossed as ‘two nines’, which uses 9 as a hapax multiplicative anchor.

*Anchor, main* An anchor is considered a system’s *main* or *primary* anchor when it is used in composing the largest number of composite numerals in the system. The main anchor of base systems is their base.

*Anchor, multiplicative* A multiplicative anchor is a number multiplied by another number to obtain the value of a composite numeral. For example, a system glossed as follows would have 5 as a multiplicative anchor for numerals for 10 and 15 (highlighted in bold): {1, 2, 3, 4, 5, 5 + 1, 5 + 2, 5 + 3, 5+4, **2⋅5,** (2⋅5) + 1, (2⋅5) + 2 …**3⋅5,** (3⋅5) + 1, (3⋅5) + 2 …}. (For numerals for 11−14 and 16−19, 5 is used as a *multiplicative–additive anchor* here). In English, 10 is used as a multiplicative anchor for all decade numbers 10−90. (For non-decade numbers between 21 and 99, English uses 10 as a *multiplicative–additive anchor*.)

*Anchor, multiplicative–additive* When the value of a composite numeral is obtained by addition operated on the product of a multiplication, the number to which that multiplication is applied is a multiplicative–additive anchor. For example, in languages like English, French, Mandarin and many others, all intra-decade numerals for numbers between 20 and 100 exploit 10 as a multiplicative–additive anchor.

*Anchor, secondary* An anchor is considered a secondary anchor when it is used in composing fewer numerals than the system’s main anchor. A special case of secondary anchor in base systems is a *sub-base*.

*Anchor, sporadic* A sporadic anchor is a number that is used in obtaining the value of fewer than half of the non-atomic numerals within a determined numerical range. A useful range to determine the status of an anchor in non-restricted systems is the *primary counting cycle* (see *counting cycle, primary* in this glossary). For example, a system glossed as follows would have 5 as a sporadic anchor (highlighted in bold): {1, 2, 3, 4, 5, 6, **5 + 2**, **5 + 3**, 9, 10, 11 …}. The Russian word for 40, ‘sorok’, is a sporadic additive anchor in the 1−100 range, since unlike other words for multiples of 10 in this range, this word has no clear decimal component, so that numerals for 41−49 are all formed by adding numbers to 40, and they are the only numerals within this range that use 40 as an anchor. A special case of sporadic anchors is hapax anchors.

*Atomic numeral* Any numeral that cannot be analysed into more than one constituent part that refers to a number is atomic. Much like atoms in physics, which can contain sub-atomic particles, atomic numerals can contain many elements, but none of them can refer to numbers. So, ‘seven’ contains five elements (i.e. five letters), but none of these elements refers to numbers. Atomic numerals can either be *free* or *bound*. Free atomic numerals (e.g. ‘seven’) can be used as numerals on their own, while bound atomic numerals (e.g. ‘teen’) cannot. For example, the French lexical numeral ‘sept’ and the Indo-Arabic numeral ‘7’ are both atomic, as is English ‘eleven’, but not ‘thirteen’, nor ‘11’. This is because the first three cannot be decomposed into parts that have numerical content, as no parts of the strings of individual letters or symbols—nor the sounds they are associated with—have numerical content. On the other hand, ‘11’ is composed of two tokens of the numeral ‘1’, and ‘thirteen’ can be analysed into two parts with numerical content: even though neither ‘thir’ nor ‘teen’ can be used on its own to refer to a specific number, both of these have numerical content, ‘thir’ being an allomorph of ‘three’ and ‘teen’ being an allomorph of ‘ten’. Similarly, a numeral that would have implicit numerical content—for example, 7 expressed as ‘and two more’ in a system where 5 is used to produce other non-atomic numerals—would not be considered atomic, since 5 could be an implicit semantic element of ‘____ and two more’. Numerals produced by *overcounting* are not atomic in the same sense, given their implicit reliance on numerical content. Atomic numerals are sometimes called ‘simplex’. Other common words for non-atomic numerals include ‘compound’, ‘composite’ and ‘complex’.

*Augend* In the operation of addition, the augend is the number *to which* another number is added. So, in *x* + *y* = *z*, *x* is the augend (*y* is the addend and *z* is the sum).

*Base* The base of a numeration system is the smallest number whose powers are used as conventionalized counting units, as manifested in the use of special representational vehicles. In other words, a numeration system only has a base when the system represents a natural number *b* in a way such that (i) powers of *b* (including *b* itself) are used as counting units and (ii) numerals for powers of *b* have distinct representational status within the system. When a system that does not satisfy (i) and (ii) has one or more numbers used to form non-atomic numerals, these numbers are called *anchors*, rather than bases. All bases are anchors with specific (exponential) properties. Not all anchors are bases. The most common base for numeration systems across representational formats is 10. Written notational systems like the Indo-Arabic and Roman numerals, spoken languages like Mandarin, and body-based systems like the Arusha finger counting system are a few examples of base 10 (i.e. decimal) systems. Aztec numerical notation is vigesimal (base 20), as is the Iqwaye body-based system. The Babylonian cuneiform positional system is base 60 with a sub-base of 10. While it is customary to consider Germanic languages like English and Romance languages like French to be decimal, it should be noted that some question this, owing to the lack of atomic numerals for some powers of 10: while the first three powers of 10 have atomic labels in such languages (e.g. ‘ten’, ‘hundred’, ‘thousand’), the fourth and fifth do not (e.g. ‘ten thousand’, ‘hundred thousand’), and then we return to an atomic label at the sixth power (e.g. ‘million’), after which a cycle of two non-atomic labels followed by an atomic one follow for each successive triad of powers of 10. For these reasons, some argue that English is a base 1000 system with a sub-base of 10, or a mixed base of 10 and 1000. However, powers of sub-bases do not receive special labels like 100 does in English. Moreover, while numerals like ‘ten thousand’ and ‘hundred thousand’ are not atomic, they still are special, in that they only contain numerals that refer to powers of 10 multiplied by other powers of 10, and they are used as conventionalized counting units. This is unlike, say, ‘three thousand six hundred’, which expresses the second power of 60 in terms of counting units that are powers of 10. This arguably supports the claim that languages like English have a layered decimal system, where the base is used in a stepwise manner.

*Compositional input, or Input* In a composite numeral, the *input* is the number used as the argument to the operation that produces the value of the non-atomic numeral. So, in ‘fourteen’, 4 is the input and 10 is the anchor, since the operation that produces the value of ‘fourteen’ is adding 4 to 10 and the more general rule in the teens here is adding a number to 10. In more complex numerals containing more than two numerals, there can be more than one compositional input, each being the number that serves as argument to the operation. The number to which the operation is applied is called the (compositional) *anchor*.

*Compositional element* A compositional element is any numeral, atomic or not, that is part of a non-atomic numeral.

*Collection (vs Set)* The term ‘collection’ can be used to refer to any group of material objects considered as a plurality (i.e. individuated under a common property). While it is common to use the term ‘set’ to describe such pluralities, sets are technical mathematical objects and as such use of the term ‘set’ may lead to confusion when talking about numbers and representations of these. To avoid adding to this confusion, terms like ‘collection’, ‘group’, ‘aggregate’, or ‘plurality’ should be used instead of ‘set’ when possible.

*Counting unit* A counting unit is a number used to count. There are two main ways we can use a number to count: as a unit from which we start a counting routine, or as a unit that determines the interval between numbers in a counting routine. In both cases, the default unit is 1. That is, we usually count by producing (lexical) numerals for 1, 2, 3, 4 and so on, in which case we start from 1 and move in intervals of 1. However, we can also count in intervals of 10, 12, 100 or in millions, billions or any other number, in which case we are using counting units larger than 1 that cover larger intervals between elements of the counting sequence. Moreover, we can use a number other than 1 as a unit *from which* to start a counting routine, in which case we are *counting on* from that number. So if we count backwards starting at 10, 9, 8 and so on, we use 10 as the counting unit from which we descend. While any number can be used as a counting unit, some counting units have special representational status in numeration systems, which is usually manifested via special signs. So, for example, speakers who use decimal lexical systems like those in English, French or Mandarin will tend to count large quantities in terms of 10 s or 100 s, rather than, say, 27 s, since their lexical systems mark 10 and operations on it in a special manner not found for numbers like 27. Those counting units that are conventionally used to perform counting operations are labelled *anchors* (see separate entry above). For more on counting units, see the contributions to this special issue by Bender [3] and by Pelland [2].

*Counting cycle* A counting cycle can be defined as a list of numerals produced for numbers between a number *n* and *f(n*), where *f(n*) typically means applying multiplication (or, more rarely addition) to *n*. Typically, for base systems, a counting cycle uses a power of a base for *n* and the next power for *f(n*). In general, starting and ending values of counting cycles are set to numerals that display a cyclical character to numeral lists, where a particular composition or sequence of numerals begins anew from a new value.

*Counting cycle, primary* The concept of a primary counting cycle is aimed at characterizing the frequency and regularity of a numeration system’s anchors within a specified range. For these purposes, the primary counting cycle can be considered a series of numerals that starts at 1 and ends at 2*n*, where *n* is the first power of the base for base systems, or the largest anchor in the system, for non-base systems. For restricted range systems, the primary counting cycle includes all numerals of the system. Having a set range for a primary counting cycle is especially useful for comparing body-based and lexical systems, which tend to be less regular than notations and object-based systems like khipus or abaci.

*Counting on (from n)* Starting a counting routine from a specifically designated number is ‘counting on’ from that number. This is often done with addition, where people may start from the larger number being added and count on from there, using the smaller one as a target, telling them how many steps to take from the larger one until they reach the value of the sum. So, if one starts counting by saying ‘ten, eleven, twelve …’, they are *counting on* from 10.

*Counting routine* A counting routine is any series of actions that, when followed, produces an ordered list of numerals, whether consecutive or non-consecutive. One can know how to produce a counting routine without knowing what numbers are and without knowing how to use the counting routine to enumerate. In this case, we can say a person is counting *intransitively* (see separate *Counting, intransitive* entry below).

*Counting unit* A counting unit is a number used to count. There are two main ways in which we can use a number to count: as a unit from which we start a counting routine, or as a unit that determines the interval between numbers in a counting routine. In both cases, the default unit is 1. That is, we usually count by producing (lexical) numerals for 1, 2, 3, 4 and so on, in which case we start from 1 and move in intervals of 1. However, we can also count in intervals of 10, 12, 100 or in millions, billions or any other number, in which case we are using counting units larger than 1 that cover larger intervals between elements of the counting sequence. Moreover, we can use a number other than 1 as a unit *from which* to start a counting routine, in which case we are *counting on* from that number. So if we count backwards starting at 10, 9, 8 and so on, we use 10 as the counting unit from which we descend. While any number can be used as a counting unit, some counting units have special representational status in numeration systems, which is usually manifested via special signs. So, for example, speakers who use decimal lexical systems like those in English, French or Mandarin will tend to count large quantities in terms of 10s or 100s, rather than, say, 27s, since their lexical systems mark 10 and operations on it in a special manner not found for numbers like 27. Those counting units that are conventionally used to perform counting operations are labelled *anchors* (see separate entry above). For more on counting units, see the contributions to this special issue by Bender and colleagues [4] and by Pelland [2].

*Counting, intransitive* Intransitive counting is the act of producing representations of numbers in order, usually in verbal form, without trying to enumerate.

*Counting on (from n)* Starting a counting routine from a specifically designated number is ‘counting on’ from that number. This is often done with addition, where people may start from the larger number and count on from there, using the smaller one as a target, telling them how many steps to take from the larger one until they reach the value of the sum. So, if one starts counting by saying ‘ten, eleven, twelve …’, they are *counting on* from 10.

*Counting, transitive* Also called *enumeration*, transitive counting is an activity whose completion allows one to determine the number of items in a collection (i.e. its cardinality) by establishing a one-to-one correspondence between a sequence of numerals and members of the collection.

*Extent* The extent of a numeration system is determined by the largest number it represents via its regular, conventional compositional rules, without requiring new signs. As Greenberg [5] noted, every language equipped with lexical numerals has a number beyond which no conventional expression exists to talk about larger numbers. For English, this is often said to be *decillion* (e.g. in Chrisomalis [6]), though less conventional words like ‘googolplex’ have been introduced to denote larger and larger numbers. Most positional notations are *unbounded* in extent, given that the compositional rules of the system include instructions for extending the system. The Indo-Arabic numerals are extendible in this sense, since no matter how large the number we represent with these numerals, adding another numeral to its left will create a new numeral representing a new larger number. For convenience, it is common to round out a system’s extent to its largest number plus 1. Following Greenberg [5], we can refer to the next number beyond a system’s extent as its *limiting number L*. Thus, for example, consider Chrisomalis’s [6] example of a limited notational system: ‘the Linear B numerals used in Mycenaean society have signs for 1, 10, 100, 1,000, and 10,000, each of which can be repeated nine times, so this system’s extent would be 100,000’ [6, p. 61]. (For more examples, see Bender *et al.* [7]. See also Chrisomalis [6 table 3.1].)

*Format of representation (or Modality of representation)* The format of a representation is determined by both its material implementation and the compositional rules that govern it. Representations can be said to have two main components: a representational *vehicle* (the physical object in which the representation is manifested) and representational *content* (what the representation is about). The *format* of a representation specifies both what sort of physical object it is (the vehicle) and what sort of rules must be followed to access the content (roughly speaking, the compositional rules, or syntax). For representations of numbers, the same signs can implement different representational formats if the rules they enter into are different. So, for example, the signs ‘0’ and ‘1’ are representational vehicles that can follow the rules of the Indo-Arabic numeration system, but they can also implement the binary system used in computing. Roman numerals and written number words can both be written on a piece of paper, but they are not in the same format, since the vehicles differ (the signs are not in the same shape) and they are not governed by the same rules. Lexical numerals follow the same combination rules (i.e. syntax) when they are spoken as when they are written down, but the vehicles are materially different, so the written and spoken numerals differ in format. Formats of numerical representations include all kinds of written symbols, knots, gestures, positions of hands and fingers, positions of beads on an abacus, bags of pebbles and number words, among many, many others. It is also common to include so-called ‘internal’ representations—e.g. patterns of neuronal activation or neuronal architectures—to describe the format of representations of number in the mind. On this view, mental representations in the brain can have different formats (e.g. analogue, propositional, perceptual, linguistic, cognitive, etc.), since different types of mental representations are processed differently and housed in different neuronal assemblies. For example, in Dehaene’s [8] highly influential triple-code model of our numerical abilities, our brain houses representations of numbers in analogue, verbal and symbolic formats. However, while it is common to talk of the brain containing representations, it should be noted that a number of theories of cognition, especially those endorsing strong versions of embodiment such as enactivism, reject brainbound representations. (See Smortchkova *et al*. [9] for more on mental representations). While the term ‘modality’ is sometimes used to describe the material implementation of a representation, use of this term risks leading to confusion, given its association with sensory modalities (e.g. sight, vision, touch, etc.). This being said, one can use ‘modality’ where there is little chance of the sensory connotation of this term leading to confusion. For an alternative take on modality, see Chrisomalis’ contribution to this special issue [10].

*Grouping (of representations of numbers)* A *grouping* is a physical manipulation of numerals that does not modify their individual shape nor the individual representational content of the grouped symbols. This is usually aimed at improving the perceptual access of the numerals taken as a group. So, for example, a simple tally expression for 12 as ‘IIIIIIIIIIII’ can be expressed via groupings as ‘IIII IIII IIII’ or as ‘IIIII IIIII II’. The term ‘chunking’ has been used in a similar way to that proposed here for ‘grouping’, but given potential confusion with the meaning of ‘chunking’ in cognitive science (e.g. Gobet *et al*. [11]), it may be best to use another term, like ‘grouping’, for cases of physical re-arrangement of numerals. For more on grouping in mathematical notations, see Schlimm’s monograph *Mathematical Notations* [12, pp. 16, 67].

*Lexical numeral* Any word or conventionalized linguistic expression used to represent numbers as part of a linguistic system is a lexical numeral. Not all representations of number presented in linguistic formats are lexical numerals, since some expressions refer to numbers but are not part of numeration systems dedicated to representing numbers in linguistic format. So, while the French ‘trois’ and the English ‘one million five hundred thousand and thirty-four’ are lexical numerals, ‘the square root of two’ is not, since it is not part of a numeration *system*. Lexical representations of numbers can be written, spoken, read (via eyes or hands) or signed.

*Multiplicand* In the operation of multiplication, the multiplicand is the number that is multiplied *by* another number. So, in *x* ⋅ *y* = *z*, *y* is the multiplicand (*x* is the multiplier and *z* is the product).

*Multiplier* In the operation of multiplication, the multiplier is the number that is multiplying another number. So, in *x* ⋅ *y* = *z*, *x* is the multiplier (*y* is the multiplicand and *z* is the product).

*N****-****ary (of a numeration system)* A system is *n*-ary (e.g. binary, quinary, decimal, vigesimal, etc.) when it contains an anchor *n* that is used frequently and regularly to construct non-atomic numerals. If a system has *n* as its base, it is *n*-ary. A non-base system can be labelled ‘*n*-ary’ when its main anchor is not a sporadic anchor, i.e. when it contains an anchor that is used in at least half of the system’s composite numerals. Since it is not always possible to determine which anchor (if any) is the system’s main anchor, and since many systems include more than one sporadic anchor, use of ‘*n*-ary’ to talk about systems themselves without qualifications can lead to confusion and is thus generally not recommended, unless it is uncontroversial what the main anchor is. Systems that only contain sporadic anchors should not be called ‘*n*-ary’, so as to better distinguish them from more regular systems. Rather, for sporadic anchors, using a variant of ‘*n*-ary’ is recommended to describe the behaviour of specific anchors *within numeral ranges* of a system. For example, French uses a decimal anchor to form most of its numerals, since it is a base 10 system, but some versions of French also use vigesimal compositional rules for a restricted range of numerals between 80 and 100. So, in these versions of French, French has a decimal system, but exploits vigesimal compositional rules in a restricted range.

*Numeral* Any representation of number that is produced by following the rules of a numeration system, regardless of format or composition (atomic or non-atomic), is a numeral.

*Number (vs numeral)* Despite efforts by many philosophers of mathematics (especially following Frege [13]), there is no universally accepted definition of numbers, and even less one that does not rely on logical concepts for its expression (e.g. Dedekind–Peano axioms). Whatever they are, numbers are not typically thought of as being material entities, although some thinkers (e.g. Brouwer and, perhaps more controversially, Mill) can be read as endorsing psychologism about numbers, according to which they are mental entities. Numbers are typically thought of as abstract discrete magnitudes: they are discrete because they can be clearly distinguished from one another, unlike elements of continua. They are magnitudes because they are typically thought to have certain sizes, which we can compare. They are abstract in that no arrangement of matter seems to constitute a number. For most, this means we cannot perceive (i.e. see, hear, touch, etc.) numbers, but we can see, hear, or touch *representations of* numbers—i.e. numerals. To avoid confusion, it may be preferable in many cases to speak of perceiving *numerosities* (see entry below) instead of numbers, when meaning to describe perception of quantities of discrete objects.

*Numerosity (vs number)* Numerosity is the number of things in a perceivable collection of physical objects. The term ‘numerosity’ was first introduced by S.S. Stevens in 1939 [14]. Since then, the status of numerosity has been unclear. Despite this ambiguity, talking about numerosity instead of number is common in the study of numerical cognition, as it helps authors across many disciplines remain agnostic about the ontological status of numbers, which is a philosophically loaded question. Roughly speaking, the difference between numerosity and number is that numerosity can be applied to an individual’s perception of numerical magnitude (i.e. the cardinality) of a physical collection of things, while numbers themselves do not have to be spoken of in relation to physical collections nor anyone’s interaction with them. There is consensus among philosophers (though not unanimity, of course) that we cannot perceive abstract objects, including numbers. But there seems to be a sense in which we can perceive, at least approximately, how many things are present in a collection (say, a dozen dots on a screen). So, to avoid saying we perceive numbers, it is common to say we perceive numerosities. Numerosity, in this sense, is a property of a stimulus. Of course, this terminological practice also raises questions about the ontological status of numerosities, motivating some philosophers to consider ‘numerosity’ as ‘a hedge term used to apply to number-like properties’[15, p. 472].

*Restricted range system* A numeration system is considered as being restricted in range when it has a low extent—that is, when it only has numerals for a comparatively small range of numbers. While there is no agreed-upon limit beyond which a numeration system ceases to be restricted, a system is typically considered restricted if it lacks conventionalized numerals for numbers larger than 5, or, following Comrie’s [16] more generous assessment in the domain of linguistics, for numbers larger than 20. For more on restricted systems, see the contributions to this special issue by Verkerk *et al*. [17], Zariquiey *et al*. [18] and Bowern [19].

*Signary (of a numeration system)* A numeration system’s signary contains all the atomic numerals to which the system’s symbol-formation rules apply. In this sense, the signary is the system’s inventory of atomic signs. For example, for Indo-Arabic numerals, the signary is {0, 1, 2, 3, 4, 5, 6, 7, 8, 9}.

*Sub-anchor (of a numeration system)* Sub-anchors are to non-base systems what sub-bases are to base systems: they are used systematically and have a specific arithmetical relation with the main anchor of the system. More precisely, a sub-anchor has to be a divisor of the main anchor, and the system must give special status to the number obtained by multiplying the main anchor by the sub-anchor, for example, by giving it an atomic label and using it as a counting unit. For example, in the Chisanbop finger calculating system [20], four fingers of the right hand each have the value of 1, while the thumb has the value of 5. The left hand is divided similarly, but each finger has the value of 10, while the thumb represents 50. Since this system cannot represent numbers beyond 99, it is not a base 10 system. However, 10 is its main anchor, and 5 is a divisor of 10 that is used systematically to compose numerals, and the multiplication of 5 by 10 has a special status (on the left thumb), so 5 is a sub-anchor in this system. Fig. 9 of Schlimm’s contribution to this special issue [21] represents the structure of this system using an abacus representation. A special case of sub-anchors is sub-bases (see entry below).

*Sub-base (of a numeration system)* In a numeration system with a base, if a secondary anchor *s* has a numerical value below the base *b*, it will be called a sub-base only if: (i) the base is a multiple of this number (or, equivalently, *s* is a divisor of *b*); (ii) there is a conventionalized counting unit above the base that is a multiple of *b* and *s;* (iii) the powers of *s* above 1 do not structure the system (say, by having special representational vehicles); and (iv) *s* is used systematically throughout the system (i.e. in the same way between powers of the base). Sub-bases are not bases, because their powers are not labelled with special representational vehicles, unlike bases. Sub-bases only occur in systems with bases. A well-known example of sub-bases is 5 in Roman numerals, where the base (10) is represented by ‘X’, and the sub-base (5) is represented by ‘V’. In this system (i) the sub-base 5 is a divisor of the base 10; (ii) multiples of 5 and 10 (e.g. 50, 500) are used as conventionalized counting units; (iii) powers of 5 (e.g. 25, 125, 625) do not have special representational status; but (iv) 5 is used in the same way between many powers of the base. Numbers that play compositional roles within base systems that do not satisfy (i)–(iv) are *secondary anchors*. For example, despite there being compositions using 20 in French (e.g. ‘quatre-vingt’, glossed as ‘four twenties’, to represent 80), 20 is not a sub-base in French, since there is no counting unit above 10 whose value is a multiple of 10 and 20. Rather, in French 20 is merely a sporadic secondary anchor (or, more precisely a sporadic secondary multiplicative–additive anchor). For more on sub-bases, see the contributions to this special issue by Chrisomalis [10] and by Schlimm [21].

*Transparency* A numeration system (or a subset of its numerals) can be considered transparent when the structure of its composite numerals mirrors the arithmetical operations used to compose its numerals. Numeration systems can lack transparency in many ways, including by having different atoms for the same number and by changing the order of components in composite numerals. For example, the numerals of languages like Mandarin, whose expression for 31 can be translated as ‘three ten one’, are considered more transparent than those of English, since ‘thirty-one’ contains neither ‘three’ nor ‘ten’. For more on transparency, see the contributions to this special issue by Göbel *et al.* [22], Chrisomalis [10], Holt & Barner [23] and Koile & Blasi [24].

.

## Data Availability

This article has no additional data.
